# Development of a questionnaire to evaluate female fertility care in pediatric oncology, a TREL initiative

**DOI:** 10.1186/s12885-022-09450-2

**Published:** 2022-04-25

**Authors:** M. E. Madeleine van der Perk, Eglė Stukaitė-Ruibienė, Žana Bumbulienė, Goda Elizabeta Vaitkevičienė, Annelies M. E. Bos, Marry M. van den Heuvel-Eibrink, Jelena Rascon

**Affiliations:** 1 Princess Máxima Center for Pediatric Oncology, Utrecht, The Netherlands; 2 Vilnius University, Faculty of Medicine, Vilnius, Lithuania; 3 Center of Obstetrics and Gynaecology, Vilnius University Hospital Santaros Klinikos, Vilnius, Lithuania; 4 University Medical Center Utrecht, Reproductive Medicine and Gynaecology, Utrecht, The Netherlands; 5 Center for Pediatric Oncology and Hematology, Vilnius University Hospital Santaros Klinikos, Vilnius, Lithuania

**Keywords:** Fertility care, Late effects, Pediatric cancer, Questionnaire, Reproductive health

## Abstract

**Background:**

Currently the five-year survival of childhood cancer is up to 80% due to improved treatment modalities. However, the majority of childhood cancer survivors develop late effects including infertility. Survivors describe infertility as an important and life-altering late effect. Fertility preservation options are becoming available to pre- and postpubertal patients diagnosed with childhood cancer and fertility care is now an important aspect in cancer treatment. The use of fertility preservation options depends on the quality of counseling on this important and delicate issue. The aim of this manuscript is to present a questionnaire to determine the impact of fertility counseling in patients suffering from childhood cancer, to improve fertility care and evaluate what patients and their parents or guardians consider good fertility care.

**Methods:**

Within the framework of the EU-Horizon 2020 TREL project, a fertility care evaluation questionnaire used in the Netherlands was made applicable for international multi-center use. The questionnaire to be used at least also in Lithuania, incorporates patients’ views on fertility care to further improve the quality of fertility care and counseling. Results evaluate fertility care and will be used to improve current fertility care in a national specialized pediatric oncology center in the Netherlands and a pediatric oncology center in Lithuania.

**Conclusion:**

An oncofertility-care-evaluation questionnaire has been developed for pediatric oncology patients and their families specifically. Results of this questionnaire may contribute to enhancement of fertility care in pediatric oncology in wider settings and thus improve quality of life of childhood cancer patients and survivors.

**Supplementary Information:**

The online version contains supplementary material available at 10.1186/s12885-022-09450-2.

## Introduction

Currently the five-year survival rate of childhood cancer is up to 80% in most European countries due to improved treatment regimens [1, 2]. However, these treatments may result in multiple long term adverse health effects such as infertility [3–7]. Impaired fertility, infertility and early menopause are highly ranked on the list of relevant side effects affecting quality-of-life in cancer survivors [8, 9]. Long-term survival after treatment for childhood cancer is associated with increased risk of impaired quality-of-life and higher prevalence of psychosocial problems often related to infertility issues [8, 9]. Fertility is thus recognized as a critical component of quality of life in young cancer survivors. Therefore, international and national guidelines recommend discussing fertility preservation (FP) before initiation of any therapy [10–14]. However, studies have shown that the majority of childhood cancer survivors (CCSs) report they had not received relevant information about reproductive health, do not know their fertility status and perceive the reproductive counseling during and after the gonadotoxic treatment as insufficient [15, 16]. Parents and patients prefer to be informed on fertility risks and preservation possibilities soon after the diagnosis, as early discussion could lead to improved quality of life, improved coping with the cancer diagnosis, cancer treatment and possible infertility, and improved social well-being, irrespective of the risk or possibilities for preservation [17–26]. Fertility counseling has revealed a beneficial impact on the quality of life after cancer treatment, regardless of the decision to preserve fertility or not [19, 23].

Adequate fertility counseling for girls with cancer comprises of individualized future fertility risk assessment and communication as well as provision of strategies to preserve gonadal material in order to maintain maximal fertility potential. This has been integrated in the Dutch amendment of the Edinburgh criteria “Standard of Cancer Care for fertility preservation” [27–29]. New fertility preservation options have become available in the past years and the importance of timely triage on gonadal damage risk, subsequent provision of information and counseling has been recognized by both patients, parents and healthcare providers [30]. Currently, oocyte cryopreservation is available for a small subset of pubertal patients who can postpone their treatment at least 2 weeks for oocyte harvest. For the majority of girls receiving high risk therapy the only available option is ovarian tissue cryopreservation. Some patients receiving radiotherapy to the pelvis can opt for a transposition of the ovaries. The American Society of Clinical Oncology has published three clinical practice guidelines with evidence-based recommendations for fertility preservation for patients with childhood cancer [8, 11, 31]. A study of compliance with these recommendations reported, however, that none of the patients above the age of 13 had been counseled for fertility preservation [32, 33]. Recently published guidelines by the International Late Effects of Childhood Cancer Guideline Harmonization Group (IGHG) advise that all patients should be informed on their potential risk of gonadal damage and should be offered counseling on fertility preservation options [34–36].

However, it is unknown how patients experience the fertility care and to date no validated questionnaires exist to evaluate this in a pediatric cancer setting. We intent to improve oncofertility care and evaluate what patients consider adequate fertility care including the impact of receiving information regarding reproductive health and fertility counseling towards fertility preservation in childhood cancer patients. Both onco-fertility care and fertility preservation methods for girls are considered standard of care since publication of the ASRM statement and IGHG guidelines [30, 36, 37]. Contrastingly, pre-pubertal male fertility preservation techniques are still considered experimental [35]. Therefore, this manuscript focusses on female fertility care. This will be evaluated using an oncofertility-care-evaluation questionnaire, initially developed at the Princess Máxima Center for Pediatric Oncology, Utrecht, The Netherlands. The Twinning in Research and Education to Improve Survival in Childhood Solid Tumours in Lithuania (TREL) is an EU-Horizon 2020 funded project that aims to improve different aspects of childhood cancer care (including survivorship care). This is done through an extensive collaboration between Vilnius University Hospital Santaros Klinikos (VULSK, Lithuania) and research intensive project partners. Implementation of Work package 6 (WP6) of the TREL project will allow to extend the oncofertility quality assessment to Lithuania. Thus, an oncofertility-care-evaluation questionnaire, which is currently used in the Netherlands was adapted for international multicenter use and in particular in Lithuania in order to improve fertility care in two pediatric oncology centers as part of the **P**reserving ov**AR**ian function through cryopr**E**servation and informing gir**L**s with cancer about infertility due to gonadotoxic treatment (PAREL) study and the TREL initiative. The aim of this manuscript is to present an oncofertility-care-evaluation questionnaire. The questionnaire aims to determine the impact of receiving information and fertility counseling in childhood cancer patients and their parents/guardians and evaluate what they consider good fertility care. This insight may be used to improve fertility care.

## Methods

### Design of the questionnaire

The questionnaire for evaluation of fertility care for girls, currently used in the Netherlands, is based on multiple validated questionnaires concerning decision regret, reproduction concern and the evaluation of fertility care in an adult setting. Relevant sections of these questionnaires were combined in the new questionnaire. Additionally, some questions were amended to fit the pediatric oncology setting and some new questions were developed. The questions from the Decision regret scale by Brehaut et al. [38] and the decisional conflict scale by O’Connor [39] were used to evaluate regret patients have concerning challenges they face in decision-making. The Dutch Reproductive Concern Scale (RCS-NL (*Voortplanting Bezorgdheid schaal*)) by Garvelink et al. [40] was used for questions regarding the patients’ concerns about infertility. We based questions concerning experiences with the fertility care on the patient-centeredness questionnaire-infertility (PCQ-infertility), which has been developed for subfertile couples [41].

The questionnaire is divided in 5 sections. The first section includes general questions to evaluate how worried patients and parents were about fertility at the time of diagnosis, whether they could recall having a conversation about fertility, and whether they proactively asked for this information. The second section contains questions concerning the first conversation regarding fertility with the nurse practitioner or the pediatric oncologist and focusses on timing and clarity of the information. The third section contains questions regarding the counseling with respect to timing and content, the knowledge on the personal risk of gonadal damage, as well as risks and benefits of the fertility preservation options. Questions regarding emotions of the patients and parents and feelings of control are also included. The fourth section contains questions regarding perceived knowledge on infertility following the information and emotions concerning the information. The last section consists of 4 open questions regarding improvement of fertility care.

The initial questionnaire was developed at the Princess Máxima Center and contains 41 items. It is given to all girls who received counseling by a fertility-gynecologist and participate in the PAREL study. The PAREL study has been approved by the Medical Ethics Committee Utrecht (METC nr. NL72115.041.19). To make it applicable for multicenter use within the TREL framework, and in particular in Lithuania, the questions were translated from Dutch to English and afterwards from English to Lithuanian (Supplemental texts 1–3). To validate the Lithuanian translation the reverse translation from Lithuanian to English was performed. No significant discrepancies between the wordings occurred. The Lithuanian version was reviewed by two pediatric oncologists, a gynecologist, two patients and parents, who were all native speaker Lithuanian and all spoke and understood English. Lastly, the Lithuanian version was compared to the Dutch version with help of the English translation by a native Dutch-speaking author.

Given the existing differences in patient numbers and the current fertility counseling system, the questionnaire was adapted to the Lithuanian situation to assess the situation of fertility counseling at VULSK within the framework of collaboration with the TREL initiative. This questionnaire contained 43 items. A separate Lithuanian questionnaire for girls who did not receive counseling by a fertility-gynecologist was created and contained 31 items. The adjustments from the Dutch to the Lithuanian version are summarized in Supplemental Table 1. The separate Lithuanian questionnaire for girls who did not receive counseling is summarized in Supplemental Table 2. The questionnaire regarding the quality of fertility counseling is currently used for all families after oncofertility counseling in the Princess Máxima Center in the Netherlands as part of the PAREL study [30]. The adapted version will be used in Lithuania for all parents and children ≥14 years old who are currently undergoing treatment or in remission for less than 5 years and who are regularly followed up at the VULSK.

#### Use of the questionnaire in two pediatric cancer centers

##### Princess Máxima Center (The Netherlands)

Since May 2018 all pediatric cancer care has been centralized in one national pediatric cancer center, the Princess Máxima Center. Around 600 children are newly diagnosed with pediatric cancer in the Netherlands every year. A 5-step oncofertility care plan is implemented since 2019 [30]. These 5 steps are 1) identification of all newly diagnosed patients, 2) triage of patients for fertility risk, 3) information provision, 4) offering counseling to a selected subgroup and 5) offer fertility preservation techniques to those at high risk of infertility, as previously described [30]. Patients are triaged on their risk of gonadal damage at the moment of diagnosis and subsequently informed by their pediatric oncologist or a dedicated oncofertility nurse practitioner. We use the developed triage table to estimate the cyclophosphamide equivalent dose (CED) score and radiation to the gonads [30]. Patients are classified as low, intermediate or high risk of infertility. The CED scores are classified as low (≤4000 mg/m^2^), intermediate (4000–6000 mg/m^2^) or high risk (≥6000 mg/m^2^) of gonadal damage [36]. However, also age at diagnosis and expected radiation to the ovaries are taken into account to estimate a personalized risk for every patient. The subset of high and intermediate risk patients is actively encouraged to go to the fertility specialist for counseling, but also low risk patients can be referred for counseling upon request. Those who are referred for counseling are given the questionnaire three to 6 months after the counseling.

##### Center for Pediatric Oncology and Hematology at Vilnius University Hospital Santaros Klinikos (VULSK) (Lithuania)

The TREL consortium is formed by VULSK and 8 leading research institutions each covering different areas of the project activities according to their expertise in pediatric oncology. TREL will be delivered in 7 work packages (WP) addressing training in tumour specific laboratory research and clinical trials, cross-cutting education on genome-wide sequencing and treatment innovations, enhancing skills in observational studies on the quality of survivorship including fertility preservation and research methodology as well as project and innovation management. TREL is a European twinning effort that aims to strengthen research networking and education in Lithuania with the ultimate goal to improve survival and quality of life of children with solid tumours (brain tumours, neuroblastoma and renal tumours). The development of the questionnaire is part of WP6 of the TREL collaboration. WP6 specifically focusses on the quality of survivorship and late effects research.

In Lithuania the questionnaire will be implemented at the Center for Pediatric Oncology and Hematology (CPOH) at VULSK, which is the biggest pediatric oncology and hematology center in Lithuania and the Baltic region. VULSK covers two thirds of pediatric cancer patients in Lithuania. Children aged from 1 month to 18 years are treated at the VULSK, every year 50–60 new patients with childhood cancer are diagnosed and treated. Approximately 50 patients and 20 survivors are currently in treatment or in remission for less than 5 years and are regularly followed up at the VULSK. At the moment, fertility counseling at VULSK is rather sporadic, gonadal tissue preservation is available after a consultation with qualified fertility specialists, but there is no developed fertility care system in place. In Lithuania the preservation of reproductive tissue is embedded in the national legislation and can be offered only to children over 14 years old. A triage system similar to the one used in the Princess Máxima Center is being developed to stratify patients according to their risk for infertility/gonadal damage [30]. Patients will be informed by the pediatric oncologist and referred to a gynecologist or urologist. VULSK aims to hand out the questionnaires three to 6 months after counseling or diagnosis. All patients will be classified as low, intermediate or high risk at the moment of diagnosis. Taking into account lower total number of patients in VULSK, the questionnaire for girls will be handed out to boys too. No changes are needed since the questions are not female specific. A developed table for boys to estimate the infertility risk by calculating CED score will be used [35].

## Discussion

The increasing number of CCSs is a reason why research is increasingly focusing on their well-being. They are at risk for infertility, which affects quality of life. As reported in a previous study on reproductive health of Lithuanian CCSs [42], many of them point out that they receive insufficient information about the impact of cancer treatment on fertility and possible preservation options. Discussing the risk for infertility with pediatric cancer patients and their parents/guardians before the gonadotoxic treatment is crucial. This paper describes the adaptation of a fertility care evaluation questionnaire for children with cancer, currently used in the Netherlands for multicenter use applicability. This is part of the collaborative effort of two TREL partners with the aim to enhance fertility care in pediatric oncology settings with a wider perspective.

It is well known that patients and parents do not remember all of the given information in stressful situations. Some studies even suggest that only 20% of the given verbal information is retained [43, 44]. In order to improve fertility counseling of childhood cancer patients, an evaluation of the current quality of fertility care will be performed using a questionnaire. To adjust the content of the information to the patient’s needs, we need to know what they consider to be important. However, no suitable questionnaire for this population existed. Therefore, we developed the current questionnaire and have implemented it in two countries. Even though, published reports suggest that patients and parents prefer this information at the time of diagnosis, for some tumour types this is not feasible [17–26]. The best timing of giving information is different for every patient e.g. in most renal tumour patients the risk of infertility can only be determined after nephrectomy, which is 4–6 weeks after diagnosis and treatment with chemotherapy in the SIOP RTSG protocol [30, 45]. Also most children with acute lymphoblastic leukemia are assigned to a treatment arm after the first 4 weeks of induction chemotherapy [30]. Therefore, a patient-tailored decision, based on international evidence and expert-based guidelines can be made to determine the timing of discussing gonadal damage (Supplemental Table S3) [30, 34–36].

Since fertility care is structured differently in the Netherlands and Lithuania, the Lithuanian questionnaire was adjusted to the local situation, e.g. a nurse practitioner is not available in the Lithuanian health system. Also the patient population will be slightly different, since a proportion of VULSK patients who receive a questionnaire may not have received oncofertility counseling by experts. In comparison, all patients receiving the questionnaire in the Netherlands have received fertility counseling from fertility experts. Bearing in mind the different cultural backgrounds, different legislations and different system of fertility counseling of childhood cancer patients in two different countries, it could be expected that the answers to the same questions may vary. This may reveal cultural differences that may influence future fertility care strategies.

## Conclusion

Oncofertility counseling is an important part of pediatric cancer care, yet no questionnaire to evaluate this existed for the pediatric population. The developed questionnaire to evaluate oncofertility care in two countries may provide insight in the views of patients and their family on offered fertility care and on improvements that could be made. Results of this questionnaire may contribute to enhanced oncofertility settings in pediatric oncology departments in a wider range of cultural and geographic settings, thereby improving quality of life of childhood cancer patients and survivors.

## Supplementary Information

**Additional file 1.**

## Data Availability

All questionnaires generated during this study are included in this published article [and its supplementary information files]. For further questions, the corresponding author can be contacted.

## Abbreviations

CCSs: Childhood cancer survivors
CED: Cyclophosphamide equivalent dose
CPOH: Center for Pediatric Oncology and Hematology
FP: Fertility preservation
IGHG: International Late Effects of Childhood Cancer Guideline Harmonization Group
PAREL: Preserving ovARian function through cryoprEservation and informing girLs with cancer about infertility due to gonadotoxic treatment
PCQ-infertility: Patient-centeredness questionnaire-infertility
RCS-NL: Dutch Reproductive Concern Scale
TREL: Twinning in Research and Education to Improve Survival in Childhood Solid Tumours in Lithuania
VULSK: Vilnius University Hospital Santaros Klinikos
WP6: Work package 6
