# Supplementary material for: Development of a questionnaire to evaluate female fertility care in pediatric oncology, a TREL initiative
Source: BMC Cancer. 2022 Apr 25;22:450. doi: 10.1186/s12885-022-09450-2 (PMC9036799; doi:10.1186/s12885-022-09450-2)
Supplement: Supplementary file 1 — Additional file 1. [file 12885_2022_9450_MOESM1_ESM.pdf]

Supplemental material

Development of a questionnaire to evaluate female fertility care in pediatric oncology, a TREL initiative.

M.E. Madeleine van der Perk and Eglė Stukaitė-Ruibienė, Žana Bumbulienė, Goda Elizabeta Vaitkevičienė, Annelies M.E. Bos, Marry M. van den Heuvel-Eibrink and Jelena Rascon

Content:

Text S1a: English questionnaire PMC (counseled)
Text S1b: Dutch questionnaire (counseled)
Text S2a: English questionnaire VULSK (counseled)
Text S2b: Lithuanian questionnaire (counseled)
Text S3a: English questionnaire VULSK (not counseled)
Text S3b: Lithuanian questionnaire (not counseled)
Table S1: Questions for counseled patients from validated questionnaires and comparing the two: VULSK and PMC
Table S2: Questions of the Lithuanian questionnaire for not-counseled children:
Table S3: Remaining challenges

S1a: English questionnaire PMC (counseled)

Questionnaire PAREL study

PAREL number:

Information and counseling conversation oncofertility

Your child was diagnosed with childhood cancer. Shortly after the diagnosis, the oncofertility team (nurse practitioner) and pediatric oncologist talked to you about the effect of treatment on your child's fertility. Afterwards, you had a counseling with the gynecologist. We would like to use this questionnaire to find out how you experienced the conversations.

Below are statements related to the conversations. You can choose from 6 options for each statement. Please indicate to what extent you agree or disagree with these statements by circling the number next to the answer that applies to you. In this way, you are indicating what you think about the decision you made. If you cannot remember, circle the statement I don't know.

Some general questions:

		Completely disagree	Disagree	Neutral	Agree	Fully agree	I don't know
1	My doctor had mentioned fertility at the first consult when the diagnosis of my child was discussed.	1	2	3	4	5	0
2	When I heard the diagnosis and treatment I was worried about my child's fertility.	1	2	3	4	5	0
3	I had to ask for information about fertility myself.	1	2	3	4	5	0

Questions about the first conversation regarding fertility with the nurse practitioner

		Completely disagree	Disagree	Neutral	Agree	Fully agree	I don't know
4	I felt it was important at that time to receive the information about fertility.	1	2	3	4	5	0
5	I thought that the moment this was discussed was a good one.	1	2	3	4	5	0
6	I thought the supporting material used during the explanation was clarifying.	1	2	3	4	5	0
7	The information about infertility was comprehensive and clear.	1	2	3	4	5	0
8	I missed important things during the conversation.	1	2	3	4	5	0

Questions regarding the counseling with the gynaecologist

		Completely disagree	Disagree	Neutral	Agree	Fully agree	I don't know
9	I think the moment of fertility counseling was a good one.	1	2	3	4	5	0
10	I thought the supporting material used during the explanation was clarifying.	1	2	3	4	5	0
11	I know the risk of infertility after treatment.	1	2	3	4	5	0
12	I was told what treatment options are available to maintain fertility.	1	2	3	4	5	0
13	The benefits of fertility preservation treatments were discussed.	1	2	3	4	5	0
14	The disadvantages of fertility preservation treatments were discussed.	1	2	3	4	5	0
15	I had control over my child's future fertility.	1	2	3	4	5	0
16	The explanation of infertility was clear.	1	2	3	4	5	0
17	The different treatment options were discussed with me.	1	2	3	4	5	0
18	The information about the treatment options was comprehensive.	1	2	3	4	5	0
19	My caregivers were honest and clear about what we could expect from the care.	1	2	3	4	5	0
20	I missed important things during the counseling.	1	2	3	4	5	0
21	I still have questions about fertility after the counseling.	1	2	3	4	5	0
22	If I have questions about fertility in the future, I know how to request another counseling.	1	2	3	4	5	0
23	Decision-making was shared with me, concerning my daughters treatment.	1	2	3	4	5	0
24	I am choosing without pressure from others	1	2	3	4	5	0

Questions after both conversations regarding fertility

		Completely disagree	Disagree	Neutral	Agree	Fully agree	I don't know
25	I am well informed about fertility.	1	2	3	4	5	0
26	I now know enough about women's fertility.	1	2	3	4	5	0
27	I know the risk of infertility from my daughters treatment.	1	2	3	4	5	0
28	I know which options are available to maintain my child's fertility.	1	2	3	4	5	0
29	I know the benefits of fertility preservation treatments.	1	2	3	4	5	0
30	I know the disadvantages of fertility preservation treatments.	1	2	3	4	5	0
31	I am angry because my child's possibilities to have children has been impaired.	1	2	3	4	5	0
32	I think it's important that my child can be a mother in the future.	1	2	3	4	5	0
33	I can talk openly about my concerns concerning my child's fertility.	1	2	3	4	5	0
34	I am sad because my daughters possibilities to have children has been impaired.	1	2	3	4	5	0
35	I made the right decision about fertility	1	2	3	4	5	0

	preservation.						
36	I regret the decision I made.	1	2	3	4	5	0
37	I would make a different decision now.	1	2	3	4	5	0

Are there things you missed during the first conversation regarding fertility or counseling?

What time do you think is the best time to have a conversation about fertility?

What did you think went well during fertility care?

Do you have any other additions or tips for fertility care?

S1b: Dutch questionnaire (counseled)

Prinses Máxima Centrum kinderoncologie

Informatie en counseling gesprek vruchtbaarheid

Uw kind kreeg de diagnose kinderkanker. De verpleegkundig specialist of kinderoncoloog heeft kort na de diagnose met u gesproken over het effect van de behandeling op de vruchtbaarheid van uw kind. Daarna heeft u nog een gesprek gehad met de gynaecoloog. Wij willen met deze vragenlijst onderzoeken hoe u het gesprek ervaren heeft.

Hieronder staan uitspraken die te maken hebben met de gesprekken. U kunt per uitspraak uit 6 opties kiezen. Geef aan hoezeer u het met deze uitspraken eens of oneens bent door het cijfer te omcirkelen bij het antwoord dat op u van toepassing is. Op deze manier geeft u aan wat u vindt van de beslissing die u genomen heeft. Als u het zich niet kunt herinneren, omcirkel dan de uitspraak weet ik niet.

Een aantal algemene vragen:

		Geheel mee oneens	Oneens	Niet mee oneens, niet mee eens	Eens	Geheel mee eens	Weet ik niet
1	De behandelaar van mijn kind had vruchtbaarheid bij het eerste gesprek toen de diagnose werd verteld al benoemd.	1	2	3	4	5	0
2	Toen ik de diagnose en behandeling hoorde, maakte ik mij zorgen om vruchtbaarheid van mijn kind.	1	2	3	4	5	0
3	Ik heb zelf om informatie over vruchtbaarheid moeten vragen.	1	2	3	4	5	0

Vragen over het eerste informatiegesprek met de verpleegkundig specialist

		Geheel mee oneens	Oneens	Niet mee oneens, niet mee eens	Eens	Geheel mee eens	Weet ik niet
4	Ik vond het belangrijk op dat moment de informatie over vruchtbaarheid te horen.	1	2	3	4	5	0
5	Ik vond het moment dat dit besproken werd een goed moment.	1	2	3	4	5	0
6	Ik vond het ondersteunende materiaal dat tijdens de uitleg gebruikt werd verduidelijkend.	1	2	3	4	5	0
7	De uitleg over onvruchtbaarheid was duidelijk.	1	2	3	4	5	0
8	Ik heb belangrijke dingen gemist tijdens het gesprek.	1	2	3	4	5	0

Vragen over het counselingsgesprek met de gynaecoloog

		Geheel mee oneens	Oneens	Niet mee oneens, niet mee eens	Eens	Geheel mee eens	Weet ik niet
9	Ik vond het moment van counseling een goed moment.	1	2	3	4	5	0
10	Ik vond het ondersteunende materiaal dat tijdens de uitleg gebruikt werd verduidelijkend.	1	2	3	4	5	0
11	Ik weet wat het risico voor mijn kind is op onvruchtbaarheid door de behandeling.	1	2	3	4	5	0
12	Mij werd verteld welke mogelijkheden er zijn om vruchtbaarheid van mijn kind te behouden.	1	2	3	4	5	0
13	De voordelen van vruchtbaarheidsbehoudende behandelingen zijn besproken.	1	2	3	4	5	0
14	De nadelen van vruchtbaarheidsbehoudende behandelingen zijn besproken.	1	2	3	4	5	0
15	Ik heb controle gehad over de toekomstige vruchtbaarheid van mijn kind.	1	2	3	4	5	0
16	De uitleg over onvruchtbaarheid was duidelijk.	1	2	3	4	5	0
17	Ik heb uitleg gekregen over de behandelmogelijkheden.	1	2	3	4	5	0
18	De uitleg over de behandelmogelijkheden was duidelijk.	1	2	3	4	5	0
19	Mijn behandelaars waren eerlijk en duidelijk over wat ik kon verwachten van de zorg.	1	2	3	4	5	0
20	Ik heb belangrijke dingen gemist tijdens het gesprek.	1	2	3	4	5	0
21	Ik heb na dit gesprek nog steeds vragen over vruchtbaarheid.	1	2	3	4	5	0
22	Als ik in de toekomst vragen heb over vruchtbaarheid van mijn kind, weet ik hoe ik opnieuw een gesprek kan aanvragen.	1	2	3	4	5	0
23	Er was ruimte om mee te beslissen over mijn dochters behandeling.	1	2	3	4	5	0
24	Ik heb kunnen kiezen zonder druk of beïnvloeding van anderen.	1	2	3	4	5	0

Vragen na afloop van beide gesprekken

25	Ik ben goed geïnformeerd over vruchtbaarheid van mijn kind.	1	2	3	4	5	0
26	Ik weet nu voldoende over vruchtbaarheid van de vrouw.	1	2	3	4	5	0
27	Ik weet wat het risico voor mijn kind is op onvruchtbaarheid door de behandeling.	1	2	3	4	5	0
28	Ik weet wat de mogelijkheden zijn om de vruchtbaarheid van mijn kind te behouden.	1	2	3	4	5	0
29	Ik weet welke voordelen er zijn van vruchtbaarheidsbehoudende behandelingen.	1	2	3	4	5	0
30	Ik weet welke nadelen er zijn van vruchtbaarheidsbehoudende behandelingen.	1	2	3	4	5	0
31	Ik ben boos omdat bij mijn kind de mogelijkheid om kinderen te krijgen is aangetast.	1	2	3	4	5	0
32	Ik vind het belangrijk dat mijn kind later	1	2	3	4	5	0

	moeder kan zijn.						
33	Ik kan openlijk praten over mijn zorgen rondom de vruchtbaarheid van mijn kind.	1	2	3	4	5	0
34	Ik ben verdrietig omdat bij mijn kind de mogelijkheid om kinderen te krijgen is aangetast.	1	2	3	4	5	0
35	Ik heb de juiste beslissing gemaakt over vruchtbaarheidsbehoud.	1	2	3	4	5	0
36	Ik heb spijt van de beslissing die ik heb gemaakt.	1	2	3	4	5	0
37	Ik zou nu een andere beslissing maken.	1	2	3	4	5	0

Zijn er dingen die u heeft gemist tijdens de gesprekken?

Welk moment is volgens u het beste moment voor een gesprek over vruchtbaarheid?

Wat vond u goed gaan tijdens de zorg rondom vruchtbaarheid?

Heeft u nog meer aanvullingen of tips voor de zorg rondom vruchtbaarheid?

S2a: English questionnaire VULSK (counseled)

Twinning in Research and Education to improve survival in Childhood Solid Tumours in Lithuania

TREL

Ver 4.0 2021-06-25

Vilnius University Hospital Santaros Klinikos is participating in Horizon 2020 project „Twinning in Research and Education to improve survival in Childhood Solid Tumours in Lithuania (TREL) Nr.952438".The aim of the project – to improve outcomes and quality of life of children diagnosed with cancer.

After the cancer treatment your child's fertility could be impaired. One of the aims of the project – to evaluate and improve the quality of fertility counseling.We would like to use this questionnaire to find out how you experienced the counseling. I am: (x)

Mother of child ☐ Father of child ☐

I raise:

Girl ☐ Boy ☐

Your child risk for infertility after childhood cancer treatment:

Low ☐ Intermediate ☐ High ☐ I don't know ☐

Statements below are related with fertility counseling. Please indicate to what extent you agree or disagree with these statements by circling one number next to the answer that applies to you (1 – completely disagree, 5 – strongly agree).

General questions:

		Completely disagree	Disagree	Neutral	Agree	Fully agree
1	My doctor had mentioned fertility at the first consult when the diagnosis of my child was discussed.	1	2	3	4	5
2	When I heard the diagnosis and treatment I was worried about my child's fertility.	1	2	3	4	5
3	I had to ask for information about fertility myself.	1	2	3	4	5

Questions about the first conversation regarding fertility with the pediatric oncologist

		Completely disagree	Disagree	Neutral	Agree	Fully agree
4	I felt it was important at that time to receive the information about fertility.	1	2	3	4	5
5	I thought that the moment this was discussed was a good one.	1	2	3	4	5
6	I received supportive material on fertility (leaflets, books, website links etc.)	1	2	3	4	5

This project has received funding from the European Union's Horizon 2020 – Work Programme on Spreading Excellence and Widening Participation under grant agreement No 952438. "

7	I thought the supporting material used during the explanation was clarifying.	1	2	3	4	5
8	The information about infertility was comprehensive and clear.	1	2	3	4	5
9	I missed important things during the conversation.	1	2	3	4	5

Questions regarding the counseling with the gynaecologist/urologist

		Completely disagree	Disagree	Neutral	Agree	Fully agree
10	I think the moment of fertility counseling was a good one.	1	2	3	4	5
11	I received supportive material on fertility (leaflets, books, website links etc.)	1	2	3	4	5
12	I thought the supporting material used during the explanation was clarifying.	1	2	3	4	5
13	I know the risk of infertility after treatment.	1	2	3	4	5
14	I was told what treatment options are available to maintain fertility.	1	2	3	4	5
15	The benefits of fertility preservation treatments were discussed.	1	2	3	4	5
16	The disadvantages of fertility preservation treatments were discussed.	1	2	3	4	5
17	I had control over my child's future fertility.	1	2	3	4	5
18	The information about infertility was comprehensive.	1	2	3	4	5
19	The different treatment options were discussed with me.	1	2	3	4	5
20	The information about the treatment options was comprehensive.	1	2	3	4	5
21	My caregivers were honest and clear about what we could expect from the care.	1	2	3	4	5
22	I missed important things during the counseling.	1	2	3	4	5
23	I still have questions about fertility after the counseling.	1	2	3	4	5
24	If I have questions about fertility in the future, I know how to request another counseling.	1	2	3	4	5
25	Decision-making was shared with me, concerning my child treatment.	1	2	3	4	5
26	I am choosing without pressure from others	1	2	3	4	5

Questions after both conversations regarding fertility

		Completely disagree	Disagree	Neutral	Agree	Fully agree
27	I am well informed about fertility.	1	2	3	4	5
28	I now know enough about fertility.	1	2	3	4	5
29	I know the risk of infertility from my child's treatment.	1	2	3	4	5
30	I know which options are available to maintain my child's fertility.	1	2	3	4	5
31	I know the benefits of fertility preservation treatments.	1	2	3	4	5

This project has received funding from the European Union's Horizon 2020 – Work Programme on Spreading Excellence and Widening Participation under grant agreement No 952438. "

32	I know the disadvantages of fertility preservation treatments.	1	2	3	4	5
33	I am angry because my child's possibilities to have children has been impaired.	1	2	3	4	5
34	I think it's important that my child can be a parent in the future.	1	2	3	4	5
35	I can talk openly about my concerns concerning my child's fertility.	1	2	3	4	5
36	I am sad because my child's possibilitiesto have children has been impaired.	1	2	3	4	5
37	I made the right decision about fertility preservation.	1	2	3	4	5
38	I regret the decision I made.	1	2	3	4	5
39	I would go for a different choice if I had to do it over again.	1	2	3	4	5

Are there things you missed during the first conversation regarding fertility or counseling?

What time do you think is the best time to have a conversation about fertility?

This project has received funding from the European Union's Horizon 2020 – Work Programme on Spreading Excellence and Widening Participation under grant agreement No 952438. "

What do you think went well during fertility care?

Do you have any other additions or tips for fertility care?

Thank you for your time!

This project has received funding from the European Union's Horizon 2020 – Work Programme on Spreading Excellence and Widening Participation under grant agreement No 952438. "

S2b: Lithuanian questionnaire (counseled)

Twinning in Research and Education to improve survival in Childhood Solid Tumours in Lithuania

TREL

Versija 3.0 2021-06-25

Vilniaus universiteto ligoninėje Santaros klinikose vyksta Horizon 2020 „Mokslinių tyrimų ir švietimo bendradarbiavimo projektas, siekiant pagerinti vaikų, sergančių piktybiniais navikais, išgyvenamumą Lietuvoje (TREL) Nr.952438“. Projekto tikslas – pagerinti vaikų, sergančių onkologinėmis ligomis išgyvenamumą ir gyvenimo kokybę pasveikus.

Pasveikus nuo onkologinės ligos gali nukentėti vaisingumas – t .y. galimybė susilaukti vaikų. Vienas iš projekto tikslų – įvertinti, ar pacientų tėvai/globėjai ir patys pacientai tinkamai informuojami apie nevaisingumo riziką.

Prieš kurį laiką Jūsų vaikui buvo diagnozuota onkologinė liga. Prašytume pasidalinti savo patirtimi užpildant šį klausimyną, siekiant pagerinti konsultavimo dėl vaisingumo kokybę.

Aš esu: (pažymėkite ☒)

Vaiko mama ☐ Vaiko tėtis ☐

Auginu:

Mergaitę ☐ Berniuką ☐

Jūsų vaiko nevaisingumo rizika po onkologinės ligos gydymo:

Žema ☐ Vidutinė ☐ Aukšta ☐ Nežinau ☐

Žemiau esantys teiginiai apibūdina konsultaciją dėl Jūsų vaiko vaisingumo. Pasirinkite vieną variantą prie kiekvieno teiginio. Nurodykite, kaip stipriai sutinkate su pateiktu teiginiu, apibraukdami skaičių prie atsakymo (1- visiškai nesutinku, 5 – visiškai sutinku).

Bendri klausimai:

		Visiškai nesutinku	Nesutinku	Nei sutinku, nei nesutinku	Sutinku	Visiškai sutinku
1	Mūsų gydytoja paminėjo vaisingumą pirmoje konsultacijoje, kai buvo aptarta mano vaiko diagnozė.	1	2	3	4	5
2	Kai sužinojau apie diagnozę ir gydymą, susirūpinau dėl savo vaiko vaisingumo.	1	2	3	4	5
3	Turėjau pats(-i) paprašyti suteikti informaciją apie vaisingumą.	1	2	3	4	5

This project has received funding from the European Union's Horizon 2020 – Work Programme on Spreading Excellence and Widening Participation under grant agreement No 952438. "

Klausimai, susiję su vaikų onkologo konsultacija:

		Visiškai nesutinku	Nesutinku	Nei sutinku, nei nesutinku	Sutinku	Visiškai sutinku
4	Jaučiau, kad tuo metu buvo svarbu gauti informaciją apie vaisingumą.	1	2	3	4	5
5	Manau, kad pokalbis apie vaisingumą įvyko tinkamu metu.	1	2	3	4	5
6	Man buvo pateikta pagalbinė medžiaga apie vaisingumą (lankstinukai, knygos, nuorodos internete ar kt.)	1	2	3	4	5
7	Pateikta pagalbinė medžiaga buvo suprantama ir aiški.	1	2	3	4	5
8	Informacija apie nevaisingumą man buvo suprantama.	1	2	3	4	5
9	Man trūko svarbių dalykų per pokalbį dėl vaisingumo.	1	2	3	4	5

Klausimai, susiję su ginekologo/urologo konsultacija:

		Visiškai nesutinku	Nesutinku	Nei sutinku, nei nesutinku	Sutinku	Visiškai sutinku
10	Manau, kad konsultacija įvyko tinkamu metu.	1	2	3	4	5
11	Man buvo pateikta pagalbinė medžiaga apie vaisingumą (lankstinukai, knygos, nuorodos internete ar kt.)	1	2	3	4	5
12	Pateikta pagalbinė medžiaga buvo suprantama ir aiški.	1	2	3	4	5
13	Aš žinau, kokia yra nevaisingumo rizika mano vaikui pasibaigus gydymui.	1	2	3	4	5
14	Man buvo papasakota, kokie yra galimi būdai vaisingumo išsaugojimui.	1	2	3	4	5
15	Buvo aptarti vaisingumo išsaugojimo gydymo privalumai.	1	2	3	4	5
16	Buvo aptarti vaisingumo išsaugojimo gydymo trūkumai.	1	2	3	4	5
17	Aš galėjau spręsti dėl savo vaiko vaisingumo ateityje.	1	2	3	4	5

This project has received funding from the European Union's Horizon 2020 – Work Programme on Spreading Excellence and Widening Participation under grant agreement No 952438. "

18	Informacija apie nevaisingumą man buvo suprantama.	1	2	3	4	5
19	Man buvo išaiškintos mano vaiko vaisingumo išsaugojimo gydymo galimybės.	1	2	3	4	5
20	Išaiškinimas apie vaisingumo išsaugojimo gydymo galimybes buvo suprantamas.	1	2	3	4	5
21	Gydytojai buvo atviri apie tai, ko galima tikėtis iš vaisingumo priežiūros.	1	2	3	4	5
22	Man trūko svarbių dalykų per konsultaciją dėl vaisingumo.	1	2	3	4	5
23	Po konsultacijos vis dar turiu klausimų apie vaisingumą.	1	2	3	4	5
24	Jeigu turėsiu klausimų apie vaisingumą ateityje, žinau, kur kreiptis.	1	2	3	4	5
25	Galėjau priimti sprendimą dėl savo vaiko vaisingumo išsaugojimo gydymo.	1	2	3	4	5
26	Galėjau rinktis be spaudimo ar kitų daromos įtakos.	1	2	3	4	5

<u>Klausimai, susiję su abiems pokalbiais dėl vaisingumo</u>:

		Visiškai nesutinku	Nesutinku	Nei sutinku, nei nesutinku	Sutinku	Visiškai sutinku
27	Aš esu gerai informuotas(-a) apie vaisingumą.	1	2	3	4	5
28	Aš dabar žinau pakankamai apie vaisingumą.	1	2	3	4	5
29	Aš žinau, kokia mano vaiko nevaisingumo rizika dėl gydymo.	1	2	3	4	5
30	Aš žinau, kokios yra mano vaiko vaisingumo išsaugojimo galimybės.	1	2	3	4	5
31	Aš žinau apie vaisingumo išsaugojimo gydymo privalumus.	1	2	3	4	5
32	Aš žinau apie vaisingumo išsaugojimo gydymo trūkumus.	1	2	3	4	5
33	Aš pykstu, nes sumažėjo mano vaiko galimybės turėti vaikų.	1	2	3	4	5
34	Man svarbu, kad mano vaikas ateityje galėtų būti mama/tėčiu.	1	2	3	4	5

This project has received funding from the European Union's Horizon 2020 – Work Programme on Spreading Excellence and Widening Participation under grant agreement No 952438. "

35	Aš galiu atvirai kalbėti apie savo susirūpinimą dėl savo vaiko vaisingumo.	1	2	3	4	5
36	Man liūdna, nes sumažėjo mano vaiko galimybės turėti vaikų.	1	2	3	4	5
37	Aš priėmiau teisingą sprendimą dėl vaisingumo išsaugojimo.	1	2	3	4	5
38	Aš gailiuosi priimo sprendimo.	1	2	3	4	5
39	Dabar aš priimčiau kitokį sprendimą.	1	2	3	4	5

Ar yra dalykų, kurių jums trūko per pirmą pokalbį ar konsultaciją dėl vaisingumo?

Kaip manote, kada yra tinkamiausias laikas pokalbiui apie vaisingumą?

This project has received funding from the European Union's Horizon 2020 – Work Programme on Spreading Excellence and Widening Participation under grant agreement No 952438. "

Kaip manote, kas praėjo gerai per Jūsų vaiko vaisingumo priežiūrą?

Ar turite kitų pastebėjimų ar patarimų dėl vaisingumo priežiūros?

Ačiū už skirtą laiką!

This project has received funding from the European Union's Horizon 2020 – Work Programme on Spreading Excellence and Widening Participation under grant agreement No 952438. "

S3a: English questionnaire VULSK (not counseled)

Twinning in Research and Education to improve survival in Childhood Solid Tumours in Lithuania

TREL

Ver. 3.0 2021-06-25

Vilnius University Hospital Santaros Klinikos is participating in Horizon 2020 project „Twinning in Research and Education to improve survival in Childhood Solid Tumours in Lithuania (TREL) Nr.952438".The aim of the project – to improve outcomes and quality of life of children diagnosed with cancer.

After the cancer treatment your child's fertility could be impaired. One of the aims of the project – to evaluate and improve the quality of fertility counseling.We would like to use this questionnaire to find out how you experienced the counseling. I am: (x)

Mother of child ☐ Father of child ☐

I raise:

Girl ☐ Boy ☐

Your child risk for infertility after childhood cancer treatment:

Low ☐ Intermediate ☐ High ☐ I don't know ☐

Statements below are related with fertility counseling. Please indicate to what extent you agree or disagree with these statements by circling one number next to the answer that applies to you (1 – completely disagree, 5 – strongly agree).

General questions

		Completely disagree	Disagree	Neutral	Agree	Strongly agree
1	I know that 80 percent of children diagnosed with childhood cancer are curable.	1	2	3	4	5
2	I know that childhood cancer treatment could affect my child's fertility (a possibility to have children after the treatment).	1	2	3	4	5
3	I had a possibility to discuss questions regarding fertility with healthcare personnel (doctors, nurses, psychologist).	1	2	3	4	5
4	I had to ask information about fertility myself.	1	2	3	4	5
5	I got information about fertility not from healthcare personnel but from other sources (Internet, other parents etc.)	1	2	3	4	5
6	I know the risk for my child's infertility after the treatment.	1	2	3	4	5

This project has received funding from the European Union's Horizon 2020 – Work Programme on Spreading Excellence and Widening Participation under grant agreement No 952438. "

Questions regarding conversation with pediatric oncologist:

		Completely disagree	Disagree	Neutral	Agree	Strongly agree
7	Pediatric oncologist mentioned a possibility and risk for fertility impairment for my child.	1	2	3	4	5
8	Information regarding fertility was understandable and clear.	1	2	3	4	5
9	I received supportive material on fertility (leaflets, books, website links etc.)	1	2	3	4	5
10	I thought the supporting material used during the explanation was clarifying.	1	2	3	4	5
11	There was room for me to have a say during a conversation about my child's fertility.		2	3	4	5
12	I thought that the moment this was discussed was a good one.	1	2	3	4	5
13	I think the space was suitable for fertility discussion.	1	2	3	4	5
14	I think practitioners used suitable voice tone during fertility discussion.	1	2	3	4	5
15	My caregivers were honest and clear about what we could expect from the care.	1	2	3	4	5
16	Decision-making was shared with me, concerning my child's treatment	1	2	3	4	5
17	I missed important things during the conversation.	1	2	3	4	5
18	I still have questions about fertility after the conversation.	1	2	3	4	5
19	If I have questions about fertility in the future, I know how to request another conversation.	1	2	3	4	5

This project has received funding from the European Union's Horizon 2020 – Work Programme on Spreading Excellence and Widening Participation under grant agreement No 952438. "

Questions regarding conversation with pediatric oncologist:

		Completely disagree	Disagree	Neutral	Agree	Strongly agree
20	I now know enough about fertility.	1	2	3	4	5
21	I know which options are available to maintain my child's fertility.	1	2	3	4	5
22	I know the benefits of fertility preservation treatment.	1	2	3	4	5
23	I know the disadvantages of fertility preserving treatment.	1	2	3	4	5
24	I think it's important that my child can be a parent in the future.	1	2	3	4	5
25	I am angry because my child's possibilities to have children has been impaired	1	2	3	4	5
26	I am sad because my child's possibilities to have children has been impaired.	1	2	3	4	5
27	I can talk openly about my concerns concerning my child's fertility.	1	2	3	4	5

Are there things you missed during the conversations regarding fertility or counseling?

This project has received funding from the European Union's Horizon 2020 – Work Programme on Spreading Excellence and Widening Participation under grant agreement No 952438. "

What time do you think is the best time to have a conversation about fertility?

What do you think went well during fertility care?

Do you have any other additions or tips on fertility care?

Thank you for your time!

This project has received funding from the European Union's Horizon 2020 – Work Programme on Spreading Excellence and Widening Participation under grant agreement No 952438. "

S3b: Lithuanian questionnaire (not counseled)

Twinning in Research and Education to improve survival in Childhood Solid Tumours in Lithuania

TREL

Versija 3.0 2021-06-25

Vilniaus universiteto ligoninėje Santaros klinikose vyksta Horizon 2020 „Mokslinių tyrimų ir švietimo bendradarbiavimo projektas, siekiant pagerinti vaikų, sergančių piktybiniais navikais, išgyvenamumą Lietuvoje (TREL) Nr.952438". Projekto tikslas – pagerinti vaikų, sergančių onkologinėmis ligomis išgyvenamumą ir gyvenimo kokybę pasveikus.

Pasveikus nuo onkologinės ligos gali nukentėti vaisingumas – t .y. galimybė susilaukti vaikų. Vienas iš projekto tikslų – įvertinti, ar pacientų tėvai/globėjai ir patys pacientai tinkamai informuojami apie nevaisingumo riziką.

Prieš kurį laiką Jūsų vaikui buvo diagnozuota onkologinė liga. Prašytume pasidalinti savo patirtimi užpildant šį klausimyną, siekiant pagerinti konsultavimo dėl vaisingumo kokybę.

Aš esu: (pažymėkite ☒)

Vaiko mama ☐ Vaiko tėtis ☐

Auginu:

Mergaitę ☐ Berniuką ☐

Jūsų vaiko nevaisingumo rizika po onkologinės ligos gydymo:

Žema ☐ Vidutinė ☐ Aukšta ☐ Nežinau ☐

Žemiau esantys teiginiai apibūdina konsultaciją dėl Jūsų vaiko vaisingumo. Pasirinkite vieną variantą prie kiekvieno teiginio. Nurodykite, kaip stipriai sutinkate su pateiktu teiginiu, apibraukdami skaičių prie atsakymo (1- visiškai nesutinku, 5 – visiškai sutinku).

Bendri klausimai

		Visiškai nesutinku	Nesutinku	Nei sutinku, nei nesutinku	Sutinku	Visiškai sutinku
1	Aš žinau, kad nuo onkologinės ligos pasveiksta 80 proc. vaikų.	1	2	3	4	5
2	Aš žinau, kad onkologinės ligos gydymas gali pakenkti mano vaiko vaisingumui – t. y. galimybei susilaukti vaikų baigus gydymą.	1	2	3	4	5
3	Turėjau galimybę aptarti vaisingumo klausimus su medicinos personalu (gydytojais, slaugytojomis, psichologe).	1	2	3	4	5

This project has received funding from the European Union's Horizon 2020 – Work Programme on Spreading Excellence and Widening Participation under grant agreement No 952438. "

4	Turėjau pats(-i) paprašyti suteikti informaciją apie vaisingumą.	1	2	3	4	5
5	Informaciją apie vaisingumą gavau ne iš medikų (interneto, bendraujant su kitais tėvais ir pan.).	1	2	3	4	5
6	Aš žinau, kokia nevaisingumo rizika kilo mano vaikui dėl ligos gydymo.	1	2	3	4	5

Klausimai, susiję su pokalbiu su gydytoja onkohematologe

		Visiškai nesutinku	Nesutinku	Nei sutinku, nei nesutinku	Sutinku	Visiškai sutinku
7	Gydytoja onkohematologė minėjo galimą vaisingumo pažeidimą ir jo riziką mano vaikui	1	2	3	4	5
8	Pokalbių metu pateikta informacija apie vaisingumą buvo suprantama ir aiški.	1	2	3	4	5
9	Man buvo pateikta pagalbinė medžiaga apie vaisingumą (lankstinukai, knygos, nuorodos internete ar kt.)	1	2	3	4	5
10	Pateikta pagalbinė medžiaga buvo suprantama ir aiški.	1	2	3	4	5
11	Galėjau įsiterpti ir išsakyti savo nuomonę pokalbiųų dėl vaisingumo metu.	1	2	3	4	5
12	Manau, kad pokalbiai dėl vaisingumo įvyko tinkamu metu.	1	2	3	4	5
13	Manau, kad pokalbiai dėl vaisingumo įvyko tinkamoje aplinkoje.	1	2	3	4	5
14	Manau, kad gydytojai apie vaisingumą kalbėjo tinkamu tonu.	1	2	3	4	5
15	Gydytojai buvo atviri apie tai, ko galima tikėtis iš vaisingumo priežiūros.	1	2	3	4	5
16	Galėjau priimti sprendimą dėl savo vaiko vaisingumo išsaugojimo gydymo.	1	2	3	4	5
17	Man trūko svarbių dalykų per pokalbius dėl vaisingumo.	1	2	3	4	5
18	Aš vis dar turiu klausimų apie vaisingumą.	1	2	3	4	5
19	Jeigu turėsiu klausimų apie vaisingumą ateityje, žinau, kur kreiptis.	1	2	3	4	5

This project has received funding from the European Union's Horizon 2020 – Work Programme on Spreading Excellence and Widening Participation under grant agreement No 952438. "

Klausimai po pokalbio apie vaisingumą

		Visiškai nesutinku	Nesutinku	Nei sutinku, nei nesutinku	Sutinku	Visiškai sutinku
20	Aš dabar žinau pakankamai apie vaisingumą.	1	2	3	4	5
21	Aš žinau, kokios yra mano vaiko vaisingumo išsaugojimo galimybės.	1	2	3	4	5
22	Aš žinau apie vaisingumo išsaugojimo gydymo privalumus.	1	2	3	4	5
23	Aš žinau apie vaisingumo išsaugojimo gydymo trūkumus.	1	2	3	4	5
24	Man svarbu, kad mano vaikas ateityje galėtų būti mama/tėčiu.	1	2	3	4	5
25	Aš pykstu, nes sumažėjo mano vaiko galimybės turėti vaikų	1	2	3	4	5
26	Man liūdna, nes sumažėjo mano vaiko galimybės turėti vaikų	1	2	3	4	5
27	Aš galiu atvirai kalbėti apie susirūpinimą dėl savo vaiko vaisingumo.	1	2	3	4	5

Ar yra dalykų, kurių jums trūko per pokalbius ar konsultacijas dėl vaisingumo?

This project has received funding from the European Union's Horizon 2020 – Work Programme on Spreading Excellence and Widening Participation under grant agreement No 952438. "

Kaip manote, kada yra tinkamiausias laikas pokalbiui apie vaisingumą?

Kaip manote, kas praėjo gerai per Jūsų vaiko vaisingumo priežiūrą?

Ar turite kitų pastebėjimų ar patarimų dėl vaisingumo priežiūros?

Ačiū už skirtą laiką!

This project has received funding from the European Union's Horizon 2020 – Work Programme on Spreading Excellence and Widening Participation under grant agreement No 952438. "

Table S1: Questions for counseled patients from validated questionnaires and comparing the two: VULSK and PMC

Section 1. General questions:

From validated questionnaires	PMC English	PMC Dutch	VULSK English	VULSK Lituanian
New	My doctor had mentioned fertility at the first consult when the diagnosis of my child was discussed.	1 De behandelaar van mijn kind had vruchtbaarheid bij het eerste gesprek toen de diagnose werd verteld al benoemd.	My doctor had mentioned fertility at the first consult when the diagnosis of my child was discussed.	1 Mūsų gydytoja paminėjo vaisingumą pirmoje konsultacijoje, kai buvo aptarta mano vaiko diagnozė.
Ik maak me zorgen of ik wel kinderen kan krijgen. (ENG Q not published) (RCS-NL VBS: 1) (1)	When I heard the diagnosis and treatment I was worried about my child's fertility.	2 Toen ik de diagnose en behandeling hoorde, maakte ik mij zorgen om vruchtbaarheid van mijn kind.	When I heard the diagnosis and treatment I was worried about my child's fertility.	2 Kai sužinojau apie diagnozę ir gydymą, susirūpinau dėl savo vaiko vaisingumo.
New	I had to ask for information about fertility myself.	3 Ik heb zelf om informatie over vruchtbaarheid moeten vragen.	I had to ask for information about fertility myself.	3 Turėjau pats(-i) paprašyti suteikti informaciją apie vaisingumą.

Section 2. Questions about the first conversation regarding fertility with the nurse practitioner/oncologist

From validated questionnaires	PMC English	PMC Dutch	VULSK English	VULSK Lithuanian
New	I felt it was important at that time to receive the information about fertility.	4 Ik vond het belangrijk op dat moment de informatie over vruchtbaarheid te horen.	I felt it was important at that time to receive the information about fertility.	4 Jaučiau, kad tuo metu buvo svarbu gauti informaciją apie vaisingumą.
New	I thought that the moment this was discussed was a good one.	5 Ik vond het moment dat dit besproken werd een goed moment.	I thought that the moment this was discussed was a good one.	5 Manau, kad pokalbis apie vaisingumą įvyko tinkamu metu.
Did you also receive written information apart from verbal information?<br>(PCQ: 3) (2)	-	-	I received supportive material on fertility (leaflets, books, website links etc.)	6 Man buvo pateikta pagalbinė medžiaga apie vaisingumą (lankstinukai, knygos, nuorodos internete ar kt.)
Did you also receive written information apart from verbal information?<br>(PCQ: 3) (2)<br>Was the information about the investigations you would undergo comprehensive?<br>(PCQ: 5) (2)	I thought the supporting material used during the explanation was clarifying.	6 Ik vond het ondersteunende materiaal dat tijdens de uitleg gebruikt werd verduidelijkend.	I thought the supporting material used during the explanation was clarifying.	7 Pateikta pagalbinė medžiaga buvo suprantama ir aiški.
Was the information about the investigations you would undergo comprehensive?<br>(PCQ: 5) (2)	The information about infertility was comprehensive and clear.	7 De uitleg over onvruchtbaarheid was duidelijk.	The information about infertility was comprehensive and clear.	8 Informacija apie nevaisingumą man buvo suprantama.
Did you miss any instructions from a nurse? If so, when?<br>(PCQ: 12) (2)	I missed important things during the conversation.	8 Ik heb belangrijke dingen gemist tijdens het gesprek.	I missed important things during the conversation.	9 Man trūko svarbių dalykų per pokalbį dėl vaisingumo.

Section 3. Questions regarding the counseling with the gynaecologist

From validated questionnaires	PMC	PMC Dutch	VULSK English	VULSK Lithuanian
New	I think the moment of fertility counseling was a good one.	9 Ik vond het moment van counseling een goed moment.	I think the moment of fertility counseling was a good one.	10 Manau, kad konsultacija įvyko tinkamu metu.
Did you also receive written information apart from verbal information?<br>(PCQ: 3) (2)	-	-	I received supportive material on fertility (leaflets, books, website links etc.)	11 Man buvo pateikta pagalbinė medžiaga apie vaisingumą (lankstinukai, knygos, nuorodos internete ar kt.)
Was the information about the investigations you would undergo comprehensive?<br>(PCQ: 5) (2)	I thought the supporting material used during the explanation was clarifying.	10 Ik vond het ondersteunende materiaal dat tijdens de uitleg gebruikt werd verduidelijkend.	I thought the supporting material used during the explanation was clarifying.	12 Pateikta pagalbinė medžiaga buvo suprantama ir aiški.
New	I know the risk of infertility after treatment.	11 Ik weet wat het risico voor mijn kind is op onvruchtbaarheid door de behandeling.	I know the risk of infertility after treatment.	13 Aš žinau, kokia yra nevaisingumo rizika mano vaikui pasibaigus gydymui.
Were different treatment options discussed with you?<br>(PCQ: 6) (2)	I was told what treatment options are available to maintain fertility.	12 Mij werd verteld welke mogelijkheden er zijn om vruchtbaarheid van mijn kind te behouden.	I was told what treatment options are available to maintain fertility.	14 Man buvo papasakota, kokie yra galimi būdai vaisingumo išsaugojimui.

Were you informed of any possible side-effects of the medication prescribed to you?<br>(PCS: 9) (2)<br><br>I know the benefits of each option<br>(DCS: 2) (3)	The benefits of fertility preservation treatments were discussed.	13 De voordelen van vruchtbaarheidsbehoudende behandelingen zijn besproken.	The benefits of fertility preservation treatments were discussed	15 Buvo aptarti vaisingumo išsaugojimo gydymo privalumai.
I know the risks and side effects of each option.<br>DCS 3. (3)	The disadvantages of fertility preservation treatments were discussed.	14 De nadelen van vruchtbaarheidsbehoudende behandelingen zijn besproken.	The disadvantages of fertility preservation treatments were discussed.	16 Buvo aptarti vaisingumo išsaugojimo gydymo trūkumai.
Ik heb controle gehad over mijn toekomstige vruchtbaarheid.<br>(ENG Q not published)<br>(RCS-NL VBS: 9) (1)	I had control over my child's future fertility.	15 Ik heb controle gehad over de toekomstige vruchtbaarheid van mijn kind.	I had control over my child's future fertility	17 Aš galėjau spręsti dėl savo vaiko vaisingumo ateityje.
Was the information about the investigations you would undergo comprehensive?<br>(PCQ: 5) (2)	The information about infertility was comprehensive.	16 De uitleg over onvruchtbaarheid was duidelijk.	The information about infertility was comprehensive.	18 Informacija apie nevaisingumą man buvo suprantama.
Were different treatment options discussed with you?<br>(PCQ: 6) (2)	The different treatment options were discussed with me.	17 Ik heb uitleg gekregen over de behandelmogelijkheden.	The different treatment options were discussed with me.	19 Man buvo išaiškintos mano vaiko vaisingumo išsaugojimo gydymo galimybės.
Was the information about the treatment you would receive comprehensive?<br>(PCQ: 7) (2)	The information about the treatment options was comprehensive.	18 De uitleg over de behandelmogelijkheden was duidelijk.	The information about the treatment options was comprehensive.	20 Išaiškinimas apie vaisingumo išsaugojimo gydymo galimybes buvo suprantamas.
Were caregivers honest and clear about what to expect from the fertility care service?<br>(PCQ: 14) (2)	My caregivers were honest and clear about what we could expect from the care.	19 Mijn behandelaars waren eerlijk en duidelijk over wat ik kon verwachten van de zorg.	My caregivers were honest and clear about what we could expect from the care.	21 Gydytojai buvo atviri apie tai, ko galima tikėtis iš vaisingumo priežiūros.

Did you miss any instructions from a nurse? If so, when?<br>(PCQ: 12) (2)	I missed important things during the counseling.	20 Ik heb belangrijke dingen gemist tijdens het gesprek.	I missed important things during the counseling	22 Man trūko svarbių dalykų per konsultaciją dėl vaisingumo.
New	I still have questions about fertility after the counseling.	21 Ik heb na dit gesprek nog steeds vragen over vruchtbaarheid.	I still have questions about fertility after the counseling.	23 Po konsultacijos vis dar turiu klausimų apie vaisingumą.
New	If I have questions about fertility in the future, I know how to request another counseling.	22 Als ik in de toekomst vragen heb over vruchtbaarheid van mijn kind, weet ik hoe ik opnieuw een gesprek kan aanvragen.	If I have questions about fertility in the future, I know how to request another counseling.	24 Jeigu turėsiu klausimų apie vaisingumą ateityje, žinau, kur kreiptis.
Was decision-making shared with you, if you preferred?<br>(PCQ: 23) (2)	Decision-making was shared with me, concerning my daughters treatment.	23 Er was ruimte om mee te beslissen over mijn dochters behandeling.	Decision-making was shared with me, concerning my child's treatment	25 Galėjau priimti sprendimą dėl savo vaiko vaisingumo išsaugojimo gydymo.
I am choosing without pressure from others<br>(DCS: 8) (3)	I am choosing without pressure from others	24 Ik heb kunnen kiezen zonder druk of beïnvloeding van anderen.	I am choosing without pressure from others	26 Galėjau rinktis be spaudimo ar kitų daromos įtakos.

Section 4. Questions after both conversations regarding fertility

From validated questionnaires	PMC English	PMC Dutch	VULSK English	VULSK Lithuanian
I have enough advice to make a choice. (DCS: 9) (3)	I am well informed about fertility.	25 Ik ben goed geïnformeerd over vruchtbaarheid van mijn kind.	I am well informed about fertility.	27 Aš esu gerai informuotas(-a) apie vaisingumą.
I have enough advice to make a choice. (DCS: 9) (3)	I now know enough about women's fertility.	26 Ik weet nu voldoende over vruchtbaarheid van de vrouw.	I now know enough about fertility.	28 Aš dabar žinau pakankamai apie vaisingumą.
New	I know the risk of infertility from my child's treatment.	27 Ik weet wat het risico voor mijn kind is op onvruchtbaarheid door de behandeling.	I know the risk of infertility from my child's treatment.	29 Aš žinau, kokia mano vaiko nevaisingumo rizika dėl gydymo.
I know which options are available to me (DCS: 1) (3)	I know which options are available to maintain my child's fertility.	28 Ik weet wat de mogelijkheden zijn om de vruchtbaarheid van mijn kind te behouden.	I know which options are available to maintain my child's fertility	30 Aš žinau, kokios yra mano vaiko vaisingumo išsaugojimo galimybės.
I know the benefits of each option (DCS: 2) (3)	I know the benefits of fertility preservation treatments.	29 Ik weet welke voordelen er zijn van vruchtbaarheidsbehoudende behandelingen.	I know the benefits of fertility preservation treatments.	31 Aš žinau apie vaisingumo išsaugojimo gydymo privalumus.
I know the risk and side effects of each option (DCS: 3) (3)	I know the disadvantages of fertility preservation treatments.	30 Ik weet welke nadelen er zijn van vruchtbaarheidsbehoudende behandelingen.	I know the disadvantages of fertility preserving treatments.	32 Aš žinau apie vaisingumo išsaugojimo gydymo trūkumus.
Ik ben boos omdat bij mij de mogelijkheid om kinderen te krijgen is aangetast. (ENG Q not published) (RCS-NL VBS: 5) (1)	I am angry because my child's possibilities to have children has been impaired.	31 Ik ben boos omdat bij mijn kind de mogelijkheid om kinderen te krijgen is aangetast.	I am angry because my child's possibilities to have children has been impaired	33 Aš pykstu, nes sumažėjo mano vaiko galimybės turėti vaikų.
New	I think it's important that my child can be a mother in the future.	32 Ik vind het belangrijk dat mijn kind later moeder kan zijn.	I think it's important that my child can be a parent in the future.	34 Man svarbu, kad mano vaikas ateityje galėtų būti mama/tėčiu.

Ik kan openlijk praten over mijn zorgen rondom mijn vruchtbaarheid. (ENG Q not published) (RCS-NL VBS: 6) (1)	I can talk openly about my concerns concerning my child's fertility.	33 Ik kan openlijk praten over mijn zorgen rondom de vruchtbaarheid van mijn kind.	I can talk openly about my concerns concerning my child's fertility.	35 Aš galiu atvirai kalbėti apie susirūpinimą dėl savo vaiko vaisingumo.
Ik ben verdrietig omdat bij mij de mogelijkheid om kinderen te krijgen is aangetast (ENG Q not published) (RCS-NL VBS: 8) (1)	I am sad because my child's possibilities to have children has been impaired.	34 Ik ben verdrietig omdat bij mijn kind de mogelijkheid om kinderen te krijgen is aangetast.	I am sad because my child's possibilities to have children has been impaired.	36 Man liūdna, nes sumažėjo mano vaiko galimybės turėti vaikų
It was the right decision. (Regret scale DRS: 1) (4)	I made the right decision about fertility preservation.	35 Ik heb de juiste beslissing gemaakt over vruchtbaarheidsbehoud.	I made the right decision about fertility preservation.	37 Aš priėmiau teisingą sprendimą dėl vaisingumo išsaugojimo.
I regret the choice that was made. (Regret scale DRS: 2) (4)	I regret the decision I made.	36 Ik heb spijt van de beslissing die ik heb gemaakt.	I regret the decision I made	38 Aš gailiuosi priimo sprendimo.
I would go for the same choice if I had to do it over again. (Regret scale DRS: 3) (4)	I would go for a different choice if I had to do it over again.	37 Ik zou nu een andere beslissing maken.	I would go for a different choice if I had to do it over again.	39 Dabar aš priimčiau kitokį sprendimą.

Section 5: Open questions:

1.
Eng: Are there things you missed during the first conversation regarding fertility or counseling?
NL: Zijn er dingen die u heeft gemist tijdens de gesprekken?
LT: Ar yra dalykų, kurių jums trūko per pirmą pokalbį ar konsultaciją dėl vaisingumo?

2.
Eng: What time do you think is the best time to have a conversation about fertility?
NL: Welk moment is volgens u het beste moment voor een gesprek over vruchtbaarheid?
LT: Kaip manote, kada yra tinkamiausias laikas pokalbiui apie vaisingumą?

3.
Eng: What do you think went well during fertility care?
NL: Wat vond u goed gaan tijdens de zorg rondom vruchtbaarheid?
LT: Kaip manote, kas praėjo gerai per Jūsų vaiko vaisingumo priežiūrą?

4.
Eng: Do you have any other additions or tips for fertility care?
NL: Heeft u nog meer aanvullingen of tips voor de zorg rondom vruchtbaarheid?
LT: Ar turite kitų pastebėjimų ar patarimų dėl vaisingumo priežiūros?

Table S2. Questions of the Lithuanian questionnaire for not-counseled children:

Section 1. General questions:

From validated questionnaires	VULSK English	VULSK Lituanian
New	1 I know that 80 percent of children diagnosed with childhood cancer are curable.	1 Aš žinau, kad nuo onkologinės ligos pasveiksta 80 proc. vaikų.
New	2 I know that childhood cancer treatment could affect my child's fertility (a possibility to have children after the treatment).	2 Aš žinau, kad onkologinės ligos gydymas gali pakenkti mano vaiko vaisingumui – t. y. galimybei susilaukti vaikų baigus gydymą.
New	3 I had a possibility to discuss questions regarding fertility with healthcare personnel (doctors, nurses, psychologist).	3 Turėjau galimybę aptarti vaisingumo klausimus su medicinos personalu (gydytojais, slaugytojomis, psichologe).
New	4 I had to ask for information about fertility myself.	4 Turėjau pats(-i) paprašyti suteikti informaciją apie vaisingumą.
New	5 I got information about fertility not from healthcare personnel but from other sources (Internet, other parents etc.)	5 Informaciją apie vaisingumą gavau ne iš medikų (interneto, bendraujant su kitais tėvais ir pan.).
New	6 I know the risk for my child's infertility after the treatment.	6 Aš žinau, kokia nevaisingumo rizika kilo mano vaikui dėl ligos gydymo.

Section 2. Questions about the first conversation regarding fertility with the oncologist

From validated questionnaires	VULSK English	VULSK Lithuanian
New	7 Pediatric oncologist mentioned a possibility and risk for fertility impairment for my child	7 Gydytoja onkohematologė minėjo galimą vaisingumo pažeidimą ir jo riziką mano vaikui
Was the information about the investigations you would undergo comprehensive? (PCQ: 5) (2)	8 Information regarding fertility provided was understandable and clear.	8 Pokalbių metu pateikta informacija apie vaisingumą buvo suprantama ir aiški.
Did you also receive written information apart from verbal information? (PCQ: 3) (2)	9 I received supportive material on fertility (leaflets, books, website links etc.)	9 Man buvo pateikta pagalbinė medžiaga apie vaisingumą (lankstinukai, knygos, nuorodos internete ar kt.)
Was the information about the investigations you would undergo comprehensive? (PCQ: 5) (2)	10 I thought the supporting material used during the explanation was clarifying.	10 Pateikta pagalbinė medžiaga buvo suprantama ir aiški.
Was decision-making shared with you, if you preferred? (PCQ: 23) (2)	11 There was room for me to have a say during a conversation about my child's fertility.	11 Galėjau įsiterpti ir išsakyti savo nuomonę pokalbių dėl vaisingumo metu.
New	12 I thought that the moment this was discussed was a good one.	12 Manau, kad pokalbiai dėl vaisingumo įvyko tinkamu metu.
New	13 I think the space was suitable for fertility discussion.	13 Manau, kad pokalbiai dėl vaisingumo įvyko tinkamoje aplinkoje.
How often did your physician have empathy for your emotions and your current situation? (PCQ: 26) (5)	14 I think practitioners used suitable voice tone during fertility discussion.	14 Manau, kad gydytojai apie vaisingumą kalbėjo tinkamu tonu.
Were caregivers honest and clear about what to expect from the fertility care service? (PCQ: 14) (2)	15 My caregivers were honest and clear about what we could expect from the care.	15 Gydytojai buvo atviri apie tai, ko galima tikėtis iš vaisingumo priežiūros.
Was decision-making shared with you, if you preferred? (PCQ: 23) (2)	16 Decision-making was shared with me, concerning my child's treatment	16 Galėjau priimti sprendimą dėl savo vaiko vaisingumo išsaugojimo gydymo.
Did you miss any instructions from a nurse? If so, when? (PCQ: 12) (2)	17 I missed important things during the conversation.	17 Man trūko svarbių dalykų per pokalbius dėl vaisingumo.

New	18 I still have questions about fertility after the conversation.	18 Aš vis dar turiu klausimų apie vaisingumą.
New	19 If I have questions about fertility in the future, I know how to request another conversation.	19 Jeigu turėsiu klausimų apie vaisingumą ateityje, žinau, kur kreiptis.

Section 3. Questions after the conversation regarding fertility

From validated questionnaires	VULSK English	VULSK Lithuanian
I have enough advice to make a choice.<br>(DCS: 9) (3)	20 I now know enough about fertility.	20 Aš dabar žinau pakankamai apie vaisingumą.
I know which options are available to me<br>(DCS: 1) (3)	21 I know which options are available to maintain my child's fertility	21 Aš žinau, kokios yra mano vaiko vaisingumo išsaugojimo galimybės.
I know the benefits of each option<br>(DCS: 2) (3)	22 I know the benefits of fertility preservation treatment.	22 Aš žinau apie vaisingumo išsaugojimo gydymo privalumus.
I know the risk and side effects of each option<br>(DCS: 3) (3)	23 I know the disadvantages of fertility preserving treatment.	23 Aš žinau apie vaisingumo išsaugojimo gydymo trūkumus.
New	24 I think it's important that my child can be a parent in the future.	24 Man svarbu, kad mano vaikas ateityje galėtų būti mama/tėčiu.
Ik ben boos omdat bij mij de mogelijkheid om kinderen te krijgen is aangetast. (ENG Q not published)<br>(RCS-NL VBS: 5) (1)	25 I am angry because my child's possibilities to have children has been impaired	25 Aš pykstu, nes sumažėjo mano vaiko galimybės turėti vaikų.
Ik ben verdrietig omdat bij mij de mogelijkheid om kinderen te krijgen is aangetast (ENG Q not published)<br>(RCS-NL VBS: 8) (1)	26 I am sad because my child's possibilities to have children has been impaired.	26 Man liūdna, nes sumažėjo mano vaiko galimybės turėti vaikų
Ik kan openlijk praten over mijn zorgen rondom mijn vruchtbaarheid. (ENG Q not published)<br>(RCS-NL VBS: 6) (1)	27 I can talk openly about my concerns concerning my child's fertility.	27 Aš galiu atvirai kalbėti apie susirūpinimą dėl savo vaiko vaisingumo.

Section 4: Open questions:

1.
Eng: Are there things you missed during the first conversation regarding fertility or counseling?
LT: Ar yra dalykų, kurių jums trūko per pokalbius dėl vaisingumo?

2.
Eng: What time do you think is the best time to have a conversation about fertility?
LT: Kaip manote, kada yra tinkamiausias laikas pokalbiui apie vaisingumą?

3.
Eng: What do you think went well during fertility care?
LT: Kaip manote, kas praėjo gerai per Jūsų vaiko vaisingumo priežiūrą?

4.
Eng: Do you have any other additions or tips for fertility care?
LT: Ar turite kitų pastebėjimų ar patarimų dėl vaisingumo priežiūros?

Table S3: Remaining challenges:

Challenges	Considerations
Optimal timing of offering information on gonadal damage	The subset of patients in whom the risk of gonadal damage cannot be ascertained at diagnosis, is a group that fertility programs need to address. Fertility preservation options including freezing semen, do not pose any procedural risks and do not influence future fertility potential. Therefore, we would recommend to offer all boys irrespective of their gonadal damage risk to store semen prior to the start of therapy. However, freezing ovarian or testicular tissue involves surgical intervention and may carry surgical and anesthetic risks. Additionally, in most young girls ovarian tissue cryopreservation removes 50% of their ovarian reserve to be stored. Thus offering these procedures to patients with unclear and possibly low gonadal damage risk may do more harm than good. However, we feel it is important to still inform patients about gonadal damage risk, even if this is unclear. Yet, depending on the situation of the patient, it may be beneficial to move the fertility discussion to a later moment, when there is less stress and more clarity concerning the gonadal damage risk.
Content of information for low risk patients	It is important to inform all patients regarding their risk of gonadal damage. However, it may be debated how much information should be offered to patients at low risk of infertility. The primary objective of discussing this topic is to take away uncertainty about fertility and offering relief that infertility is not something to worry about. Thus, the information can be kept to the bare minimum. However, since changes in the treatment and gonadotoxicity may occur and information is not always repeated, some additional information may be valuable. This ensures parents and children have the background information needed to realize the importance of addressing this topic again.
Timing of information for low risk patients	The diagnostic period can be a very stressful and chaotic time and given information at that time is not always remembered. For girls and pre-pubertal boys with a low risk of infertility gonadal damage is not a topic requiring immediate action. Thus, it may not be the best moment to discuss gonadal damage with these patients at diagnosis. However, since it has been reported that patients worry about fertility from diagnosis, it may be valuable to split the information into two moments. At diagnosis it may be mentioned that infertility is not expected to be a problem and that a detailed discussion will follow in the near future. This way the worry can be taken away, but also the information given at a later time. During the second moment parents and patients are expected to be in a less stressful period and expected to retain more of the given information.
Repetition of information	There may be multiple reasons to repeat the information given at diagnosis about fertility at a later time. Reasons may be that not all information is retained at that stressful time, changes in treatment or attitude regarding fertility preservation may have occurred, but also some treatments can cause some degree of memory loss and thus patients may not remember that information was given or even their personal risk of gonadal damage. By repeating the information, patients and parents are more likely to be correctly informed about their personal gonadal damage risk.

References

1. Garvelink MM, ter Kuile MM, Louwe LA, Hilders CG, Stiggelbout AM. Validation of a Dutch Version of the Reproductive Concerns Scale (RCS) in Three Populations of Women. Health Care Women Int. 2015;36(10):1143-59.
2. van Empel IW, Aarts JW, Cohlen BJ, Huppelschoten DA, Laven JS, Nelen WL, et al. Measuring patient-centredness, the neglected outcome in fertility care: a random multicentre validation study. Hum Reprod. 2010;25(10):2516-26.
3. O'Connor AM. Validation of a decisional conflict scale. Med Decis Making. 1995;15(1):25-30.
4. Brehaut JC, O'Connor AM, Wood TJ, Hack TF, Siminoff L, Gordon E, et al. Validation of a decision regret scale. Med Decis Making. 2003;23(4):281-92.
